# A mobile messaging service for families on postnatal knowledge and practices: a cluster randomized trial, India

**DOI:** 10.2471/BLT.24.292145

**Published:** 2025-02-13

**Authors:** Jamie Sewan Johnston, Pooja Suri, Shirley Yan, Adithi Chandrasekar, Saumya Singla, Victoria C Ward, Seema Murthy

**Affiliations:** a Stanford Center for Health Education, Stanford University, 408 Panama Mall, Stanford, CA 94070, United States of America (USA).; b University of California, Berkeley, USA.; c Noora Health, San Francisco, USA.; d YosAid Innovation Foundation, Bengaluru, India.; e Stanford University School of Medicine, Stanford, USA.; f Noora Health India PLC, Bengaluru, India.

## Abstract

**Objective:**

To evaluate the impact of a mobile messaging service that delivers World Health Organization recommendations on postnatal care to families from birth through six weeks postpartum via a messaging platform.

**Methods:**

We randomized tertiary hospitals in four Indian states into two groups. In the treatment group, 15 hospitals promoted the messaging service to families in maternity wards before discharge following a recent birth. Nine control hospitals provided standard in-hospital information. From mid-March 2021 to mid-January 2022, we recruited mothers to participate in the study. Consenting mothers completed a face-to-face baseline survey before hospital discharge after birth and a follow-up phone survey roughly 6 weeks postpartum. Using logistic regression controlling for state-fixed effects and baseline covariates, we examine intent-to-treat estimates and report risk differences.

**Findings:**

A total of 21 937 participants met the inclusion criteria. We observed significant positive impacts in 7 out of 11 neonatal and maternal care practices examined (*P*-values < 0.05). Breastfeeding increased by 3.1 percentage points, recommended cord care practices by 4.1 percentage points, skin-to-skin care with mothers by 9.2 percentage points, and skin-to-skin care by fathers by 2.2 percentage points. For recommended maternal dietary practices, we observed significant increases in adherence to guidelines advising no reduction of food intake (7.1 percentage points), no reduction of water intake (7.9 percentage points) and no restrictions on food items (10.8 percentage point; *P*-values < 0.01).

**Conclusion:**

This study demonstrates that concise yet comprehensive digital messaging delivered to families during the postpartum period can effectively encourage recommended postnatal care practices.

## **Objectif **Évaluer l’impact d’un service de messagerie mobile qui transmet aux familles les recommandations de l’Organisation mondiale de la santé au sujet des soins postnatals, depuis la naissance jusqu’à six semaines après l’accouchement.

**Méthodes:**

Nous avons randomisé les hôpitaux de soins tertiaires de quatre États indiens selon deux groupes. Dans le groupe de traitement, 15 hôpitaux ont fait la promotion du service de messagerie auprès des familles au sein des maternités avant la sortie de l’hôpital à la suite d’une naissance récente. Neuf hôpitaux de contrôle ont fourni des informations standard à l’hôpital. De la mi-mars 2021 à la mi-janvier 2022, nous avons recruté des mères pour participer à l’étude. Les mères qui ont accepté de participer ont répondu à une enquête de référence en face à face avant la sortie de l’hôpital après la naissance et à une enquête téléphonique de suivi environ 6 semaines après l’accouchement. Nous avons appliqué une régression logistique pour contrôler les effets fixes par État et les covariables de base et avons examiné les estimations en intention de traiter et rapportons les différences de risque.

**Résultats:**

En tout, 21 937 participantes ont répondu aux critères d’inclusion. Nous avons remarqué des impacts positifs significatifs dans 7 des 11 établissements de soins néonatals et maternels examinés (valeurs *p* < 0,05). L’allaitement maternel a augmenté de 3,1 points de pourcentage, les pratiques recommandées de soins du cordon ombilical de 4,1 points de pourcentage, les contacts peau à peau avec les mères de 9,2 points de pourcentage et les contacts peau à peau avec les pères de 2,2 points de pourcentage. En ce qui concerne les pratiques alimentaires maternelles recommandées, nous avons observé des augmentations significatives de l’application des directives conseillant de ne pas réduire l’apport alimentaire (7,1 points de pourcentage), de ne pas réduire la consommation d’eau (7,9 points de pourcentage) et de ne pas éviter la consommation de certains produits alimentaires (10,8 points de pourcentage; valeurs *p* < 0,01).

**Conclusion:**

Cette étude démontre que la diffusion par voie numérique de messages brefs mais complets aux familles pendant la période postnatale permet d’encourager efficacement les pratiques recommandées en matière de soins postnatals.

## Resumen

**Objetivo:**

Evaluar el impacto de un servicio de mensajería móvil que ofrece recomendaciones de la Organización Mundial de la Salud sobre atención posnatal a las familias desde el nacimiento hasta seis semanas después del parto a través de una plataforma de mensajería.

**Métodos:**

Se dividieron de forma aleatoria los hospitales de tercer nivel de cuatro estados de la India en dos grupos. En el grupo de tratamiento, 15 hospitales promovieron el servicio de mensajería entre las familias de las salas de maternidad antes del alta tras un parto reciente. Nueve hospitales de control proporcionaron información estándar sobre el hospital. Desde mediados de marzo de 2021 hasta mediados de enero de 2022, se reclutaron madres para participar en el estudio. Las madres que dieron su consentimiento completaron una encuesta de referencia presencial antes del alta hospitalaria tras el parto y una encuesta por teléfono de seguimiento aproximadamente 6 semanas después del parto. Mediante una regresión logística que controla los efectos fijos del estado y las covariables de referencia, se examinan las estimaciones por intención de tratar y se notifican las diferencias de riesgo.

**Resultados:**

Un total de 21 937 participantes cumplieron los criterios de inclusión. Se observaron efectos positivos significativos en 7 de las 11 prácticas de atención neonatal y materna que se examinaron (valores de *p* <0,05). La lactancia materna aumentó en 3,1 puntos porcentuales, las prácticas recomendadas de cuidado del cordón umbilical en 4,1 puntos porcentuales, el contacto piel con piel con las madres en 9,2 puntos porcentuales y el contacto piel con piel con los padres en 2,2 puntos porcentuales. En cuanto a las prácticas alimentarias maternas recomendadas, se observaron aumentos significativos en el cumplimiento de las directrices que aconsejaban no reducir la ingesta de alimentos (7,1 puntos porcentuales), no reducir la ingesta de agua (7,9 puntos porcentuales) y no restringir los alimentos (10,8 puntos porcentuales; valores de *p* <0,01).

**Conclusión:**

En este estudio, se demuestra que los mensajes digitales, concisos pero completos, que se envían a las familias durante el periodo posparto pueden fomentar de forma efectiva las prácticas de atención posnatal recomendadas.

## ملخص

الغرض تقييم تأثير خدمة الرسائل عبر الهاتف المحمول التي تقدم توصيات منظمة الصحة العالمية بشأن رعاية ما بعد الولادة للأسر، من الولادة وحتى ستة أسابيع بعد الولادة عبر منصة الرسائل.

الطريقة قمنا بتقسيم المستشفيات الجامعية في أربع ولايات هندية إلى مجموعتين. في مجموعة العلاج، قامت 15 مستشفى بالترويج لخدمة الرسائل للأسر في أقسام الولادة قبل الخروج بعد ولادة حديثة. تسعة من المستشفيات الخاضعة للمراقبة قدمت معلومات قياسية داخل المستشفى. من منتصف مارس/آذار 2021 إلى منتصف يناير/كانون ثاني 2022، قمنا بتجنيد أمهات للمشاركة في الدراسة. أكملت الأمهات المشاركين مسحًا أساسيًا وجهاً لوجه قبل الخروج من المستشفى بعد الولادة، ومسحًا هاتفيًا للمتابعة بعد حوالي 6 أسابيع من الولادة. باستخدام التحوّف اللوجستي للتحكم في التأثيرات الثابتة للولاية والمتغيرات المشتركة الأساسية، نقوم بفحص تقديرات نية العلاج والإبلاغ عن فروق المخاطر.

النتائج استوفى ما مجموعه 21937 مشاركًا معايير الإدراج. لاحظنا تأثيرات إيجابية ملموسة في 7 من أصل 11 ممارسة تم فحصها لرعاية حديثي الولادة والأمهات (قيم نسبة الاحتمال < 0.05). زادت الرضاعة الطبيعية بنسبة 3.1 نقطة مئوية، وممارسات العناية بالحبل السري الموصى بها بنسبة 4.1 نقطة مئوية، والعناية المباشرة بالجلد مع الأمهات بنسبة 9.2 نقطة مئوية، والعناية المباشرة بالجلد بواسطة الآباء بنسبة 2.2 نقطة مئوية. بالنسبة لممارسات التغذية الموصى بها للأمهات، لاحظنا زيادات ملموسة في الالتزام بالمبادئ التوجيهية التي تنصح بعدم تقليل كمية الطعام (7.1 نقطة مئوية)، وعدم تقليل كمية الماء (7.9 نقطة مئوية)، وعدم فرض قيود على المواد الغذائية (10.8 نقطة مئوية؛ قيم نسبة الاحتمال < 0.01).

الاستنتاج توضح هذه الدراسة أن الرسائل الرقمية الموجزة والشاملة المقدمة للأسر خلال فترة ما بعد الولادة، يمكنها أن تشجع بشكل فعال ممارسات رعاية ما بعد الولادة الموصى بها.

## 摘要

**目的:**

该移动信息服务通过信息平台向各家庭提供世界卫生组织关于从出生到六周内产后护理的建议，本文目的在于评估该移动信息服务的影响。

**方法:**

我们将印度四个邦的三级医院随机分为两组。治疗组中，15 家医院最近在产妇出院前向产科病房的家庭推广了该信息服务。九个对照医院提供了标准住院信息。从 2021 年 3 月中旬到 2022 年 1 月中旬，我们招募了多位母亲参与研究。同意参与的母亲在产下新生儿后出院前完成了一项面对面基线调查，并在产后大约 6 周内完成了一项随访电话调查。我们通过邦固定效应的逻辑回归控制和基线协变量检查了治疗意向估值并报告了风险差异。

**结果:**

共有 21 937 名参与者符合纳入标准。我们观察到，每 11 个新生儿和产妇护理实践案例中有 7 例具有显著的积极影响（P 值 <0.05）。母乳喂养比例提高 3.1 个百分点，脐带护理推荐做法提高 4.1 个百分点，母亲肌肤接触护理提高 9.2 个百分点，父亲肌肤接触护理提高 2.2 个百分点。对于推荐的产妇饮食习惯，我们观察到，遵循不减少食物摄入量（7.1 个百分点）、不减少水摄入量（7.9 个百分点）和不限制食物种类（10.8 个百分点；P值 <0.01）的指南建议的家庭数量显著增长。

**结论:**

本研究表明，在产后护理期间向家庭提供简洁而全面的数字信息可有效促进所推荐产后护理实践的实现。

## Резюме

**Цель:**

Оценить влияние мобильного сервиса обмена сообщениями, который предоставляет рекомендации Всемирной организации здравоохранения по послеродовому уходу семьям с момента рождения ребенка до шести недель после родов с помощью платформы обмена сообщениями.

**Методы:**

Специализированные больницы третичного звена в четырех индийских штатах были разделены на две группы. В группе лечения 15 больниц рекламировали услугу обмена сообщениями для семей в родильных отделениях перед выпиской после недавних родов. Девять контрольных больниц предоставили стандартную внутристационарную информацию. С середины марта 2021 года по середину января 2022 года проводился набор матерей для участия в исследовании. Матери, давшие согласие, прошли исследование исходного состояния перед выпиской из больницы после родов при личной встрече и последующий телефонный опрос примерно через 6 недель после родов. При использовании логистической регрессии, контролирующей фиксированные по состоянию эффекты и исходные ковариаты, были получены оценки совокупности всех рандомизированных пациентов согласно назначенному лечению и представлены различия в рисках.

**Результаты:**

Всего критериям включения соответствовали 21 937 участников. Было отмечено значительное положительное влияние в 7 из 11 рассмотренных методов ухода за новорожденными и матерями (*P*-значения < 0,05). Показатели частоты грудного вскармливания увеличились на 3,1 процентного пункта, рекомендованной практики ухода за пуповиной – на 4,1 процентного пункта, телесного контакта с матерью – на 9,2 процентного пункта, а телесного контакта с отцом – на 2,2 процентного пункта. В отношении рекомендованной привычной диеты матери наблюдалось значительное увеличение приверженности рекомендациям, предписывающим не сокращать потребление пищи (7,1 процентного пункта), не сокращать потребление воды (7,9 процентного пункта) и не ограничивать продукты питания (10,8 процентного пункта; *P*-значения < 0,01).

**Вывод:**

Результаты данного исследования свидетельствуют о том, что краткие, но всеобъемлющие цифровые сообщения, направляемые семьям в послеродовой период, могут эффективно стимулировать рекомендуемые методы послеродового ухода.

## Introduction

Reducing maternal and neonatal mortality is a global health priority, particularly in countries like India, where the burden is high.^1^^–^^3^ For the last decade, Indian national and state policy initiatives have focused on decreasing preventable maternal and infant deaths.^4^ However, despite recent progress, maternal and neonatal mortality remain high, particularly among the country’s poorest households.^1^^,^^5^^,^^6^

To save lives and improve infant and mother well-being, experts have highlighted the importance of health education interventions that improve postnatal care practices.^7^^–^^10^ The provision of predischarge information and postnatal health education to new parents in the critical time period following childbirth has been shown to improve their knowledge and care practices.^11^

Several health education interventions aim to improve the knowledge,^12^ including the care companion programme designed by the non-profit organization Noora Health in partnership with governments in Bangladesh, India and Indonesia. This in-hospital education programme addresses gaps in postnatal education in hospitals,^13^^,^^14^ by offering interactive group sessions run by health educators in postpartum maternity wards, designated to teach mothers and their family caregivers essential care practices following World Health Organization (WHO) guidelines.^15^^,^^16^ The sessions, typically 20–30 minutes in length, involve the use of flipcharts, visuals aids and videos where televisions are available.^14^

The care companion programme model is based on evidence supporting the importance of family members in postnatal care.^17^^,^^18^ In India, family members, particularly grandmothers of new infants, not only support new mothers with caregiving but are also primary household decision-makers for maternal and infant care.^19^^,^^20^ Research indicates that new mothers are more likely to adopt WHO-recommended best practices when they have strong family support.^21^^,^^22^

While the model has shown improved health outcomes,^12^^,^^13^^,^^23^ anecdotal evidence suggests gaps in the care companion programme, notably in follow-up care after discharge. Many mothers do not attend postnatal doctor appointments and miss out on essential information and support. This issue is especially common among the poorest families and those residing in rural regions, because of their limited literacy and fewer opportunities to access health centres and critical health information.^24^^,^^25^ Furthermore, health workers face challenges related to the time and resources required to deliver quality in-person postnatal education.^26^

Digital technologies present a promising opportunity to overcome these challenges. In India, smartphone penetration has greatly increased in the last decade, with social media and messaging platforms like WhatsApp (Meta, Menlo Park, United States of America) widely used across populations, including those with lower socioeconomic status.^27^ Research suggests that parents seek out infant care advice via mobile devices,^28^^,^^29^ and numerous studies have demonstrated the effectiveness of perinatal education delivered through mobile phones.^30^^–^^47^ Digital messaging interventions have the potential to bridge gaps in in-person information delivery, promoting attendance in antenatal and postnatal care visits,^30^^–^^36^ increasing breastfeeding and safe infant feeding and care,^36^^–^^38^ and improving immunization coverage.^32^^,^^33^^,^^38^^–^^42^ However, more studies are needed to establish key strategies to improve the effectiveness of mobile messaging interventions, particularly in low- and middle-income countries.^32^^–^^34^

Recognizing this opportunity accompanying the widespread adoption of smartphones in India, the Noora Health’s care companion programme includes a mobile extension of the programme, the mobile care companion programme. This extension aims to continue providing key health information directly to households through messaging platforms in the critical months following the discharge of new mothers from the hospital. In this study, we evaluate the impact of the mobile care companion programme delivered through WhatsApp on maternal and newborn care practices through a cluster randomized trial in four Indian states.

## Methods

### Intervention

The mobile care companion programme delivers continued care advice to families via a free mobile messaging service on WhatsApp. At the end of every in-person care companion training session, health educators encourage mothers and family caregivers (typically fathers and grandmothers of newborns) to enrol in the mobile programme and provide a phone number to sign up for the service.

The programme delivers 25 messages, including seven videos, over 50 days (available in the online repository).^48^ The messages align with the content of the in-person programme and promote WHO-recommended postnatal practices, including early and exclusive breastfeeding, hygienic cord care, skin-to-skin care, vaccination, maternal nutrition recommendations and recognizing warning signs of critical illness in mothers and babies.^15^^,^^16^ The messages have been reviewed by a team of medical experts and state health departments, who also piloted the messages among target learners. The service is available in seven languages: English, Hindi, Kannada, Marathi, Punjabi, Tamil and Telugu. Like the in-person programme, the mobile messages direct advice to entire families rather than exclusively to new mothers. Upon introduction to the mobile care companion programme, the programme health educators encourage family members to engage with the service alongside new mothers.

In addition to receiving care advice through the mobile messages, recipients can also ask questions on WhatsApp. A team of trained support staff monitors and responds to questions; however, they do not provide medical consultation or advice on medications, rather they direct recipients to health providers for such queries.

### Study design

To evaluate the effectiveness of the mobile care companion programme, we conducted a cluster-randomized controlled trial, with hospitals as the unit of randomization. We registered the trial on Open Science Framework.^48^ The Consolidated Standards of Reporting Trials (CONSORT)checklist is available in the online repository.^48^

Based on programmatic feasibility and resources, Noora Health determined the number of hospitals to be included in the study. To achieve a representative sample of hospitals, we first stratified districts in each state by India’s health management information health index quartiles, and randomly selected a set of high- and low-delivery load hospitals where the care companion programme had been implemented for at least two years. We selected 26 tertiary hospitals in four states (Karnataka, Madhya Pradesh, Maharashtra and Punjab) for inclusion in the study.

We excluded two hospitals from the study. One hospital was excluded before randomization because the facility shifted focus to coronavirus disease 2019 (COVID-19) treatment and redirected births to alternative facilities. The second hospital was excluded after randomization when the state government requested the hospital, originally assigned to the standard-of-care control group, begin implementing the mobile care companion programme intervention three months into the study. The hospitals were stratified by state, and 15 were randomly assigned via a random number generator to the treatment group where the mobile extension of the programme would be promoted. The remaining nine hospitals served as a control and provided only the in-person programme.

Noora Health decided to recruit and enrol participants during a 10-month period. From mid-March 2021 to mid-January 2022, data collection field teams recruited new mothers in maternity wards across the 24 hospitals during their hospital stays, following birth and before discharge. Birthing women who had not experienced a stillbirth and were present at the time of data collection were invited to participate. Inclusion criteria required completing a demographic survey at recruitment and household ownership of a smartphone. Families in which newborns or mothers died before discharge were excluded. In treatment hospitals, out of 14 018 mothers recruited, 11 611 (82.8%) consented and met inclusion criteria. In control hospitals, 10 326 (87.4%) out of 11 806 recruited consented and met inclusion criteria.

### Measures

Data collection teams conducted face-to-face demographic surveys with all consenting mothers during recruitment. If new mothers were unable to respond, family members designated by the mother responded on their behalf. We constructed a socioeconomic status index using the same method as the Indian National Family Health Survey 4, assigning pre-determined weights to each socioeconomic indicator. We then summed and standardized these weights around the control group's index mean. All consenting mothers self-identified themselves as female in the demographic survey.

About eight weeks (55 days on average) after participants consented, data collection teams attempted to contact all participants for a follow-up phone survey (online repository).^48^

Primary outcomes included self-reported post-discharge behaviours in three categories: (i) newborn care practices, including exclusive breastfeeding, any breastfeeding, recommended umbilical cord care, skin-to-skin care and six-week immunization; (ii) maternal dietary practices, including adherence to recommendations not to reduce or restrict food and water, and consumption of iron, folic acid and calcium supplements; and (iii) newborn and maternal complications, including post-discharge hospital readmissions. Secondary outcomes included knowledge of newborn care practices regarding infant feeding, skin-to-skin care and cord care. Because of the programmatic limitations in the number of clusters (hospitals) and overall sample size of the study, the study had insufficient power to examine impact on maternal and infant mortality as primary outcomes.

We controlled for baseline characteristics, obtained from the demographic survey, and imputed missing values for covariates using the median of the non-missing values conditioned on the state. The final set of covariates included in the model was determined using post-double selection,^45^ with a complete list of high-dimensional controls provided in the online repository.^48^

### Statistical analysis

To estimate the impact of the mobile care companion programme intervention, we calculated intent-to-treat estimates, which capture the effect of offering mobile messages, regardless of take-up, using the following logistic regression equation:

*log* (*P_ih_) = α*
*+ β_1_ T_h_ + γ X_ih_ + θ_s_ + ε_ih_* (1)

where the dependent variable *P_ih_* represents the probability of an outcome for a mother *i* recruited in hospital *h*. The treatment variable *T_h_* is a binary variable, which is 0 if a hospital is assigned the standard of care, and 1 if it is assigned the intervention. Our main estimate of interest is *β_1_* which provides the intent-to-treat estimate of the impact of the mobile intervention as compared to the in-person care companion programme. The vector *X_ih_* consists of mother- and hospital-level covariates, selected using post-double selection to improve precision.^45^ The intercept *α* represents the baseline log-odds of the outcome in the control group, accounting for covariates and fixed effects. We included state-fixed effects (*θ_s_*) and *ε_ih_* is the error term clustered at the hospital level. We used a logistic regression model to estimate the results as outcomes are binary and report risk differences using the Stata *margins* command. To correct for multiple hypothesis testing, we calculated sharpened false discovery rate adjusted *q*-values.^46^ Analyses were conducted in Stata version 16 (StataCorp LLC, College Station, United States of America (USA)).

### Ethics review

This study received approval from the ACE Ethics Committee (DCGI Reg. No. ECR/141/Indt/KA/2013/RR-19) based in Bangalore, India and the Institutional Review Boards at Stanford University, Stanford, USA (IRB-65931) and the University of California, Berkeley, USA (2024–04–17420). All participants consented in their local language.

## Results

### Sample

**Fig. 1:**
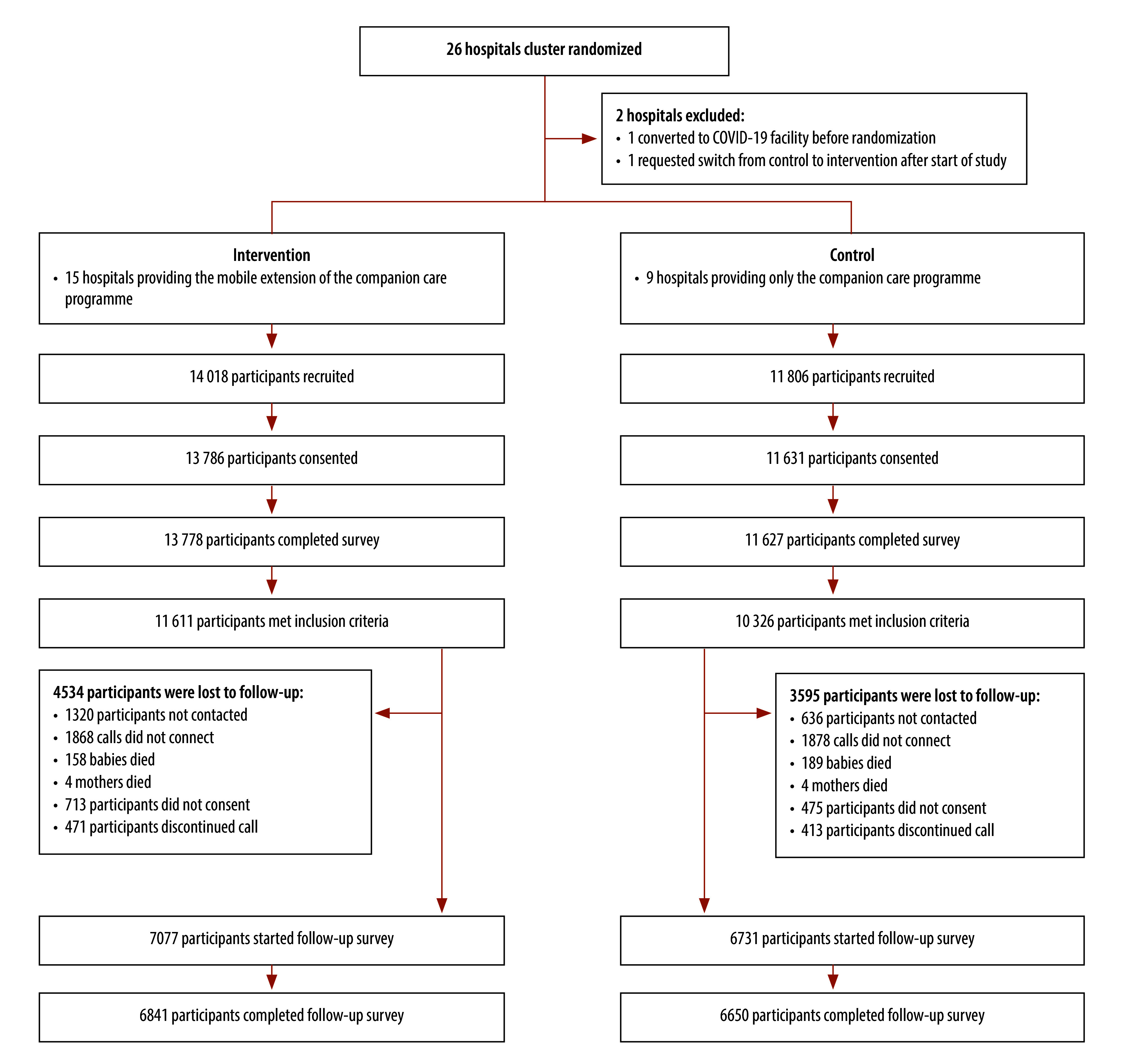
Participant flowchart for a cluster randomized trial of a postnatal mobile messaging service for families, India, 2021–2022

Of the 25 824 participants recruited for the in-hospital demographic survey, 25 417 (98.4%) consented and 25 401 (98.4%) completed the survey (Fig. 1). Of the participants who completed survey, 11 611 in the intervention group and 10 326 in the standard-of-care group met the inclusion criteria. The excluded participants had a lower socioeconomic status (−0.573: standard deviation, SD: 1.153) than participants in the control group (0.0; SD: 1.0) and the intervention group (−0.01; SD: 1.01; Table 1 and online repository).^48^

COVID-19: coronavirus disease 2019.

Notes: Participant inclusion criteria includes completion of baseline survey at the time of recruitment in hospitals and household ownership of a smartphone through which WhatsApp can be accessed. Households with mothers or babies who died before hospital discharge were also excluded.

**Table 1:** Baseline characteristics of participants in the randomized controlled trial on mobile messaging service for postnatal care practices, India, 2021–2022

Characteristic	No. (%)^a^	
	Control group (*n* = 6650)	Intervention group (*n* = 6841)
**Average mother's age in years (SD)**	24.27 (3.77)	24.39 (3.66)
**Average no. of live births (SD)**	1.29 (0.99)	1.16 (0.95)
**Newborn is male**	3476 (52.3)	3586 (52.4)
**Mother as primary respondent**	6560 (98.6)	6651 (97.2)
**Vaginal delivery**	3765 (56.6)	3431 (50.2)
**First-time mother**	1451 (21.8)	1815 (26.5)
**Social category**		
General	936 (14.1)	816 (11.9)
Scheduled caste or scheduled tribe	5467 (82.2)	5667 (82.8)
Other	247 (3.7)	358 (5.2)
**Ownership of a below poverty line card**	3810 (57.3)	4158 (60.8)
**Socioeconomic status index^b^**	0.00 (1.00)	−0.01 (1.01)
**Ownership of household item**		
Pressure cooker	5202 (78.2)	5328 (77.9)
Colour television	4976 (74.8)	4934 (72.1)
Refrigerator	2621 (39.4)	2670 (39.0)
Table	1602 (24.1)	2752 (40.2)
Washing machine	623 (9.4)	1155 (16.9)
Sewing machine	1580 (23.8)	1412 (20.6)
Air conditioner or cooler	2243 (33.7)	1821 (26.6)
Mattress	3778 (56.8)	4308 (63.0)
Motorcycle or scooter	4003 (60.2)	4063 (59.4)
None of these	337 (5.1)	431 (6.3)
**Household characteristic**		
Concrete roof	4149 (62.4)	4540 (66.4)
Uses liquefied petroleum gas mostly for cooking	5496 (82.6)	6054 (88.5)
Uses open toilet	1802 (27.1)	890 (13.0)
**Highest level of education**		
No formal schooling	824 (12.4)	365 (5.3)
Primary school	408 (6.1)	292 (4.3)
Secondary school	3091 (46.5)	3310 (48.4)
Higher than secondary school	2324 (34.9)	2865 (41.9)
**Occupation**		
Unemployed	27 (0.4)	201 (2.9)
Homemaker	5364 (80.7)	5946 (87.0)
Self-employed	241 (3.6)	255 (3.7)
Daily wage worker	862 (13.0)	246 (3.6)
Private sector employee	100 (1.5)	152 (2.2)
Public sector employee	53 (0.8)	32 (0.5)

NA: not applicable.

^a^ Value represents no. (%) unless otherwise stated.

^b^ The socioeconomic status index is calculated using the method from the Indian National Family Health Survey 4 and standardized around the control group.

Notes: Inconsistencies arise in some values due to rounding. The data represents the final analytic sample after loss-to-follow-up.

The overall loss-to-follow-up was 37.1% (Fig. 1), similar to previous postnatal follow-up phone surveys conducted by Noora Health.^13^ We observed no significant differences in attrition between the control and treatment groups (online repository).^48^ Attrition was more common among participants with the following characteristics: someone other than the mother completed the demographic survey; newborn readmission to the hospital after delivery; belonging to other minority social categories; and fewer household assets (online repository).^48^

Examining the pre-intervention characteristics of the 13 491 mothers who completed the follow-up survey showed that the intervention and control groups were well balanced (Table 1). We observed similar balance between the two randomized groups in the baseline survey (online repository).^48^ Significant, but small differences in the proportion of first-time mothers and number of previous births existed between the groups (Table 1).

The average age of all mothers was 24.33 years (SD: 3.72). Vaginal delivery accounted for just over half of the births, and mothers had an average of 1.23 previous births (SD: 0.97). Of the 13 491 mothers, most (11 134; 82.5%) belong to a scheduled caste or tribe indicating lower socioeconomic status, and most (7968; 59.1%) possessed a below poverty line card. Additionally, most mothers are homemakers (11 310; 83.8%) and completed secondary education (11 590; 85.9%).

### Participation in the intervention

Of the 6841 mothers responding to the follow-up survey, 4224 (61.7%) could recall who enrolled the household in the mobile care companion programme. Of these, 70.1% (2963) reported themselves as the household member that initiated enrolment, 22.2% (937) reported the spouses and 7.7% (324) reported other relatives as initiators of enrolment. Only 3015 (44.1%) mothers recalled viewing the mobile messages. However, of these, nearly all (2909; 96.5%) reported understanding the messages; 1131 (37.5%) recalled viewing any videos; 477 (15.8%) recalled asking questions on the platform; and 846 (28.1%) shared information or videos with other family members. Of the 1261 mothers whose family members enrolled, 848 (67.2%) recalled family members sharing information or videos with them (Table 2). The uptake of practice and knowledge reported in the follow-up survey is presented in Table 3.

**Table 2:** Mother-reported uptake and engagement in a postnatal mobile messaging service for families, India, 2021–2022

Variable	No. (%) (*n* = 6841)
**Recalled which family member enrolled in programme**	4224 (61.7)
Mother (self)	2963 (70.1)
Spouse	937 (22.2)
Other family member	324 (7.7)
**Could not recall if anyone enrolled in programme**	2617 (38.3)
**Engagement with programme**	
Mother recalled viewing messages on WhatsApp	3015 (44.1)
Understood messages received	2909 (96.5)
Viewed any linked videos	1131 (37.5)
Asked questions on the platform	477 (15.8)
Shared information or videos with family members	846 (28.1)
Family members shared information or videos with the mother^a^	848 (67.2)

^a^ Whether a family member shared the message with the mother was only asked of 1261 mothers who recalled family members enrolling in the programme.

**Table 3:** Uptake of postnatal care practices and knowledge reported in the follow-up survey of a cluster randomized trial of a postnatal mobile messaging service for families, India, 2021–2022

Outcome	No. of participants reporting an outcome (%)	
	Control group (*n* = 6650)	Intervention group (*n* = 6841)
**Practice**		
Newborn care practice		
Exclusive breastfeeding	3316 (49.9)	3310 (48.4)
Fed any breastmilk	6259 (94.1)	6737 (98.5)
Practiced recommended cord care	3255 (48.9)	4155 (60.7)
Mother practiced skin-to-skin with newborn	622 (9.4)	1209 (17.7)
Father practiced skin-to-skin with newborn	69 (1.0)	219 (3.2)
Completed six-week vaccinations	3387 (50.9)	3942 (57.6)
Maternal dietary practice		
No reduction of food intake	4635 (69.7)	5243 (76.6)
No reduction of water intake	4996 (75.1)	5380 (78.6)
No restriction of food items	2530 (38.0)	3486 (51.0)
Consumed iron and folic acid supplements	822 (12.4)	1425 (20.8)
Consumed calcium supplements	770 (11.6)	1467 (21.4)
**Newborn and maternal complication**		
Newborn admitted to hospital after discharge	453 (6.8)	407 (5.9)
Newborn experienced complications after discharge	1961 (29.5)	2041 (29.8)
Mother admitted to hospital after discharge	78 (1.2)	107 (1.6)
Mother experienced complications after discharge	745 (11.2)	859 (12.6)
Experienced issues with cord care	369 (5.5)	379 (5.5)
**Knowledge**		
Know to breastfeed when mother has a fever	2674 (40.2)	3230 (47.2)
Know to breastfeed jaundiced newborn	3560 (53.5)	4074 (59.6)
Know age to start complementary feeding	4540 (68.3)	4790 (70.0)
Know recommended cord care	2526 (38.0)	3353 (49.0)
Know about skin-to-skin care	1233 (18.5)	2191 (32.0)

### Effect on postnatal care practices

The mobile care companion programme intervention significantly improved four of the six newborn care practices measured. Mothers receiving mobile messages experienced increases of 3.1 percentage points (95% confidence interval, CI: 1.5–4.74) in feeding any breastmilk; 4.1 percentage points (95% CI: 0.4–7.8) in practicing recommended cord care; 9.2 percentage points (95% CI: 6.2–12.2) in skin-to-skin care by the mother; and 2.2 percentage points (95% CI: 0.7–3.8) in skin-to-skin care by the father. We observed no significant impact on exclusive breastfeeding or six-week vaccinations (Table 4).

**Table 4:** Effect of a postnatal mobile messaging service for families on newborn care, maternal diet, complications and knowledge, India, 2021–2022

Outcome	Mean % of participants in control group^a^	Risk difference, percentage points (95% CI)	*q* ^b^
**Practice**			
Newborn care practice			
Exclusive breastfeeding	49.9	1.8 (−6.0 to 9.6)	0.563
Fed any breastmilk	94.1	3.1 (1.5 to 4.7)	0.001
Practiced recommended cord care	48.9	4.1 (0.4 to 7.8)	0.056
Mother practiced skin-to-skin with newborn	9.4	9.2 (6.2 to 12.2)	0.001
Father practiced skin-to-skin with newborn	1.0	2.2 (0.7 to 3.8)	0.012
Completed six-week vaccinations	50.9	4.2 (−1.5 to 9.9)	0.259
Maternal dietary practice			
No reduction of food intake	69.7	7.1 (5.0 to 9.2)	0.001
No reduction of water intake	75.1	7.9 (3.6 to 12.1)	0.001
No restriction of food items	38.0	10.8 (7.1 to 14.5)	0.001
Consumed iron and folic acid supplements	12.4	6.0 (−3.2 to 15.3)	0.267
Consumed calcium supplements	11.6	6.5 (−1.6 to 14.6)	0.214
**Newborn and maternal complication**			
Newborn admitted to hospital after discharge	6.8	0.3 (−1.7 to 2.3)	0.697
Newborn experienced complications after discharge	29.5	2.2 (−0.8 to 5.1)	0.259
Mother admitted to hospital after discharge	1.2	0.2 (−0.4 to 0.8)	0.504
Mother experienced complications after discharge	11.2	2.1 (0.7 to 3.5)	0.010
Experienced issues with cord care	5.5	−0.3 (−1.5 to 0.9)	0.563
**Knowledge**			
Know to breastfeed when mother has a fever	40.2	8.7 (4.1 to 13.3)	0.001
Know to breastfeed jaundiced newborn	53.5	4.9 (−2.4 to 12.1)	0.267
Know age to start complementary feeding	68.3	−4.5 (−10.9 to 1.9)	0.265
Know recommended cord care	38.0	2.1 (−3.6 to 7.9)	0.504
Know about skin-to-skin care	18.5	14.9 (10.5 to 19.4)	0.001

CI: confidence interval.

^a^ Mean percentages of the intervention group are presented in Table 3.

^b^ *q* denotes adjusted false discovery rate to correct for multiple hypothesis testing.^41^

Notes: We ran a logistic regression of the primary outcomes on treatment for 13 491 respondents. Each row represents a separate regression. All outcome variables presented are indicators that are 0 if the behaviour was not practiced, and 1 if the behaviour was practiced. The treatment variable is an indicator that is 0 if they were assigned to the control group and 1 if they were assigned to treatment. Covariates were included in all regressions using post-double selection,^40^ and missing values were imputed using the median of the non-missing values conditional on the state. Standard errors (not shown here) are robust, and are clustered at the hospital level.

We observed significant improvements in three of the five maternal diet practices measured. Mothers in the intervention group were significantly more likely to adhere to recommendations advising that they do not reduce their food intake (7.1 percentage points increase; 95% CI: 5.0–9.2) or water intake (7.9 percentage points increase; 95% CI: 3.6–12.1) or restrict specific food items (10.8 percentage points increase; 95% CI: 7.1–14.5). We did not observe any significant impact on supplement consumption (Table 4).

We observed no differences in newborn complications at follow-up. Mothers in the intervention group were significantly more likely to report experiencing complications after discharge (2.1 percentage points; 95% CI: 0.7–3.5).

Across all care practices, we examined heterogeneity in differences for first-time mothers and young mothers (aged 18–24 years) and did not observe substantive differences in outcomes (online repository).^48^

### Effect on postnatal care knowledge

We observed a significant impact on two of the five knowledge outcomes measured. Mothers in the intervention group were significantly more likely to know that breastfeeding is recommended even when the mother has a fever (8.7 percentage points; 95% CI: 4.1–13.3) and to know about skin-to-skin care (14.9 percentage points; 95% CI: 10.5–19.4; Table 4).

Calculations of *q*-values included all outcomes reported in Table 4 and confirmed that the findings were consistent with *P*-values. The unadjusted results of the primary outcomes on treatment are robust to adjusted specifications (online repository).^48^

## Discussion

The study contributes to the mobile health messaging literature in several ways. First, it provides causal evidence on the effectiveness of a replicable model of mobile messaging in driving behaviour change and improving uptake of key recommended care practices among low socioeconomic status populations. Second, while most postnatal education interventions focus on a narrow set of practices, such as breastfeeding or immunization alone,^11^ the mobile care companion programme offers concise yet comprehensive messaging across a broad set of postnatal care practices. Third, unlike many postnatal interventions, the mobile care companion programme actively engages households by inviting all family members involved in caregiving to participate alongside new mothers. The intervention design recognizes the role of family members in postnatal care and decision-making and accommodates shared mobile phone use, which is often controlled by male heads of households.^43^^,^^44^

Our study findings add to a growing body of evidence showing that mobile dissemination of postnatal care education can improve care practices.^30^^–^^42^ By delivering concise, spaced messages over six weeks, the mobile extension of the companion care programme positively influenced the uptake of WHO-recommended postnatal care practices. While the mobile extension covered the same general topics as the in-person education provided to the control group, the addition of spaced mobile-delivered messages led to improvements in most neonatal practices measured. These findings suggest the added value of mobile messaging as a complementary tool, allowing families to access information at their convenience and retain it for future reference. 

This study further highlights the importance of delivering health education to family members involved in postnatal care. Although our study relied on self-reported survey data due to the inability to link WhatsApp platform analytics with participants, our findings show that family members initiated enrolment in nearly one third of households. In these households, two thirds of mothers reported receiving information from their family members. Similarly, nearly one third of mothers who recalled using the programme shared messages with family members. However, less than half of mothers recalled viewing the programme’s messages, likely reflecting limited smartphone access among women. Addressing shared phone ownership dynamics and increasing smartphone access for mothers could increase the effectiveness of mobile education while also challenging traditional gender norms around caregiving. Prior studies have shown that mobile messaging interventions can increase men’s involvement in caregiving, suggesting potential for broader impact.^36^^,^^40^

This study demonstrates the feasibility of achieving impact among low socioeconomic populations. The proliferation of smartphones in India, even among marginalized groups, highlights the potential of digital interventions to overcome barriers to quality in-person care. In our study, only one in eight participants were excluded due to lack of a smartphone, showing that digital tools can reach low-income mothers delivering in tertiary hospitals.

This study has some limitations. First, we only included mothers who had delivered in a tertiary hospital, possibly excluding the poorest in communities. Nonetheless, mothers participating in the study predominantly belonged to low socioeconomic status populations. Second, although the mobile care companion programme was designed as a two-way communication service, it was largely used as a one-way information delivery system, with only one in seven mothers (among mothers who recalled viewing messages) reporting that they asked questions on the platform. Because of the small number of mothers using the interactive component of the service, we were unable to assess its impact. Third, we were unable to observe how information was consumed within households. This drawback underscores the need to develop effective strategies for directing educational content to new mothers and family members. Finally, some messages were likely mistimed, arriving too late to influence caregiving practices. Tailoring messages to specific patient needs and improving the timing of delivery could increase the programme’s impact. We recommend that future studies explore how interactive messaging services can identify patient needs and provide tailored information. Future advancements in technology, such as artificial intelligence, could help improve personalization and timing of mobile messages to better meet individual needs

In conclusion, this study suggests that even simple information delivery through smartphones can positively impact care, consistent with evidence that one-way postnatal messaging can be cost-effective in saving lives.^47^ The impact observed for relatively simple messaging underscores opportunities to improve mobile interventions to improve postnatal care and ultimately save lives.
